# Beyond the Atypical: Gastrointestinal Manifestations in Kawasaki Disease

**DOI:** 10.7759/cureus.76559

**Published:** 2024-12-29

**Authors:** Arwa Ahmed, Amal Irfan Khazi, Zaineb Benslimane, Amira Ahmed, Khurshid Khan

**Affiliations:** 1 Pediatric Medicine, Al Qassimi Women’s and Children’s Hospital, Sharjah, ARE; 2 Pediatrics, Al Qassimi Women’s and Children’s Hospital, Sharjah, ARE; 3 Radiology, Al Qassimi Women’s and Children’s Hospital, Sharjah, ARE; 4 General Pediatrics, Al Qassimi Women’s and Children’s Hospital, Sharjah, ARE

**Keywords:** atypical presentation, coronary aneurysm, kawasaki disease (kd), prolonged fever, transient intussusception

## Abstract

Kawasaki disease (KD) is an acute vasculitis mainly seen in children, with a specific risk for coronary artery involvement. Atypical symptoms can sometimes result in missed diagnoses, delaying necessary treatment and increasing the chances of serious cardiovascular complications.

We report a case of a six-month-old previously healthy girl who had not been vaccinated. She presented with high fever, vomiting, and diarrhea for two weeks. The initial treatment for a suspected upper respiratory infection and later gastrointestinal issues created a diagnostic challenge. After many tests and scans over several weeks, a clear diagnosis was made only when an echocardiogram showed dilated coronary arteries and a dilated aortic root. She was then diagnosed with atypical KD and was given high doses of aspirin and intravenous immunoglobulins, resulting in substantial improvement in her condition.

This case points out the difficulty in diagnosing KD in very young children, particularly when typical signs are missing. The delayed identification of KD led to serious complications, highlighting the importance of greater awareness among healthcare workers about atypical symptoms in infants. Quick action is vital to reduce the risk of severe cardiovascular consequences and to avoid delaying necessary treatment.

## Introduction

Kawasaki disease (KD) is an acute multisystem inflammatory vasculitis that primarily affects medium-sized vessels with a particular predilection for the coronaries [1], making it a leading cause of acquired heart disease in the pediatric age group [2]. However, there is a high susceptibility for coronary vessel involvement making it a hallmark of KD. Atypical KD is a known entity in the literature. Fever for five or more days is one common finding. However, fever may be absent or missed in some infants. Patients with incomplete KD have less than four signs of mucocutaneous inflammation [3].

Fulfilling the diagnostic criteria for KD which consists of fever for at least five days accompanied by four out of five criteria, oral mucosal changes, conjunctival injection, rash, non-suppurative cervical lymphadenopathy, and changes of peripheral extremities, allows for a straightforward diagnosis [4]. However, a set of patients exhibit symptoms that do not initially meet the established criteria leading to the classification known as "Atypical Kawasaki Disease" [1,5,6]. This variant can pose significant challenges in clinical diagnosis leading to delays in intervention, development of complications, and subsequently a high risk of morbidity and mortality.

In this case report, a six-month-old infant initially presented with fever, vomiting, and diarrhea, suspected to be viral gastroenteritis. The child was treated with intravenous hydration and showed improvement, including resolution of the fever. However, persistent irritability and elevated inflammatory markers led to further investigations. A lumbar puncture was normal, but an echocardiogram revealed significant coronary artery dilation. The infant had blood-streaked stools but no major abdominal findings. Based on these symptoms, coronary dilation, and high inflammatory markers, KD was diagnosed. Treatment with IV methylprednisolone, intravenous immunoglobulin (IVIG), and aspirin was started after discussion with pediatric rheumatology, resulting in clinical improvement and reduced inflammatory markers.

A week later, the infant returned with bloody stools and irritability. An abdominal ultrasound revealed the "doughnut sign" of intussusception. After consultation with pediatric surgery, the patient was managed medically and improved within three days. The infant was discharged, and a follow-up protocol for KD was established.

## Case presentation

A six-month-old previously healthy unvaccinated Pakistani girl presented with complaints of intermittent fever, vomiting, and diarrhea for two weeks. She had been passing watery stools about five times per day with two episodes of bloody stools. During the same time frame, she also experienced coryzal symptoms along with difficulty breathing mostly during episodes of fever. There was no history of previous disease, travel, or sick contact.

On initial assessment, she was irritable and clinically dehydrated with increased capillary refill time, and her baseline activity level was noticeably affected according to her mother. Prior to the patient’s encounter with us, she was managed as an outpatient in a private GP clinic, as a case of upper respiratory tract infection where she received antibiotics including Ceftriaxone IM for two days and continued on oral amoxicillin due to persistent symptoms. Four days later at follow-up, she was prescribed metronidazole and azithromycin for five days with the clinical diagnosis of an ear infection and dysentery.

Her physical examination revealed an irritable infant with a temperature of 38.1°C, a respiratory rate of 35/min, and a heart rate ranging between 140 and 150/min. There were no rashes, lymphadenopathy, conjunctivitis, oral, or extremity changes. She was vitally stable with a heart rate of 132 beats per minute. Her saturation was 98% on room air. No signs of meningeal irritation were observed during the examination.

**Figure 1:**
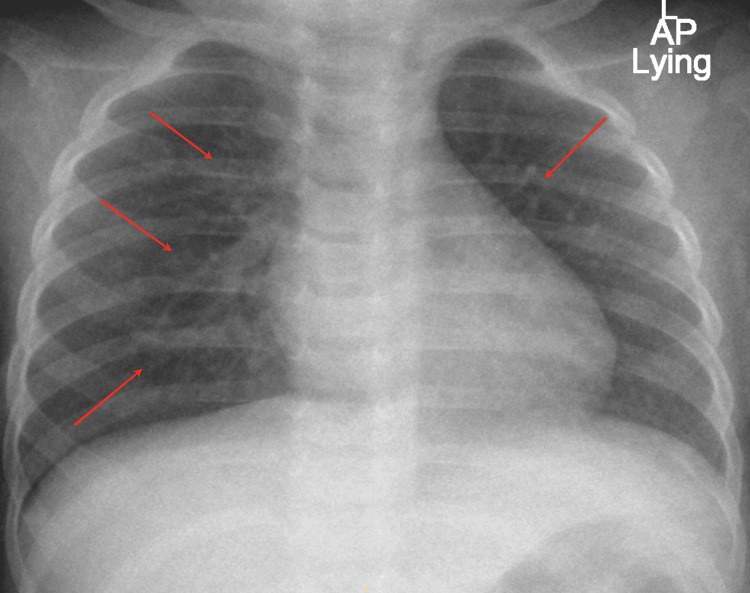
Chest X-ray showing prominent broncho-vascular markings suggesting a picture of bronchiolitis Red arrows pointing at prominent broncho-vascular markings

She was admitted as a case of prolonged fever without clear focus. Basic laboratory studies (Table 1), blood, urine, and stool cultures were sent and she was started on empirical IV ceftriaxone (75mg/kg/day). Her laboratory findings were remarkable for leukocytosis, thrombocytosis, anemia, and high C-reactive protein (CRP) (Table 1). Blood, urine, and stool cultures were negative. An abdominal and pelvic ultrasound was unremarkable. A chest X-ray showed increased broncho-vascular markings, suggesting acute bronchiolitis (Figure 1). COVID-19 and Influenza A&B PCR were negative.

**Table 1:** Laboratory findings during the first admission

Test	Day 1	Day 3	Day 7	Normal values
White blood cells	22.9 × 10^9^/L (high)	24.66 × 10^9^/L (high)	26.42 × 10^9^/L (high)	4.0 -11.0 × 10^9^/L
Hemoglobin	7.70 g/dL (low)	8.40 g/dL (low)	7.10 g/Dl (low)	12.0-16.0 g/dL
Hematocrit	25.90 % (low)	28.50% (low)	23.20 % (low)	36-46%
C-reactive protein	122 mg/dL (high)	112 mg/dL (high)	213 mg/dL (high)	< 3.0 mg/dL

**Figure 2:**
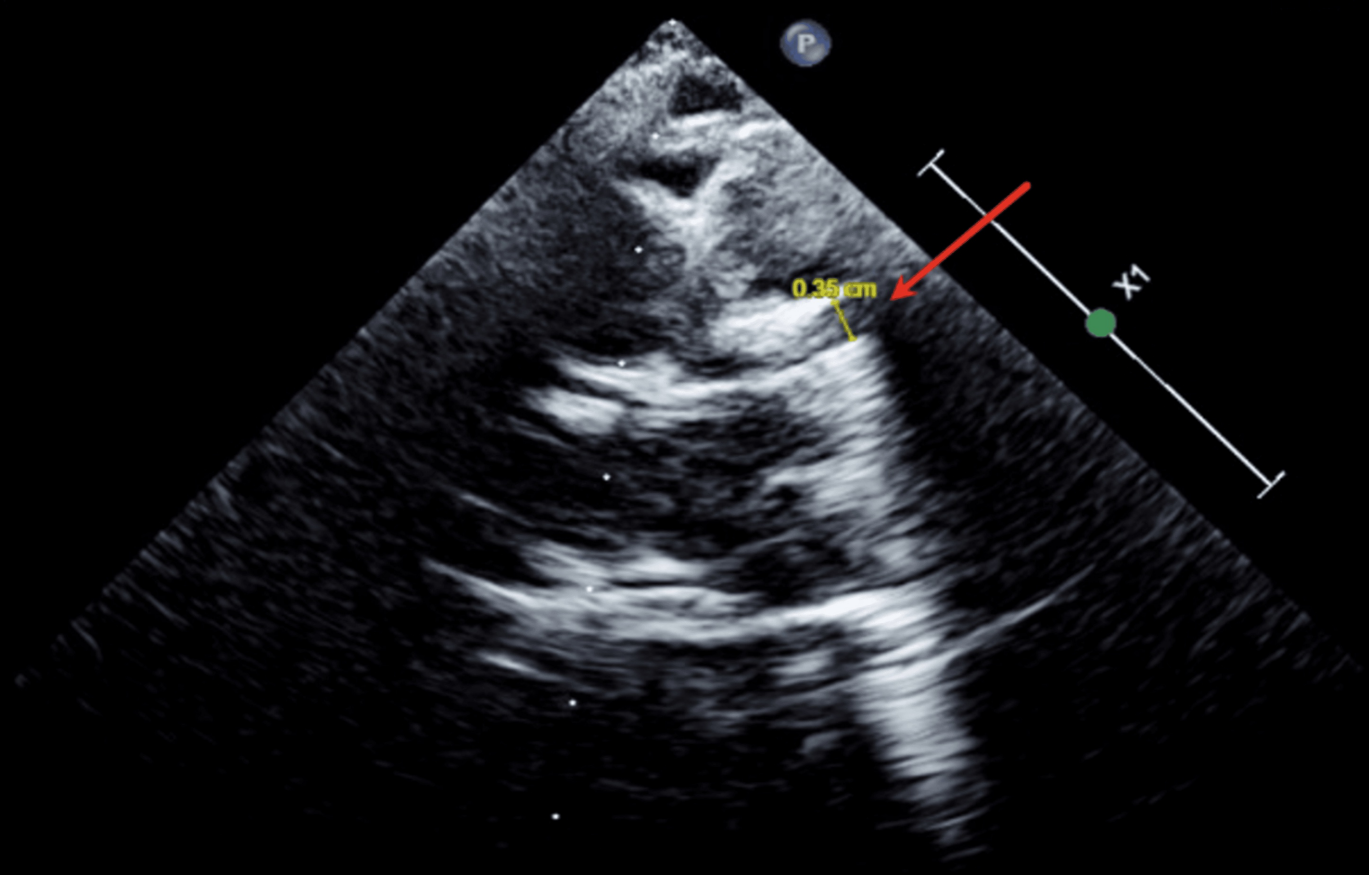
Echocardiogram from the parasternal long-axis view showing a dilated left anterior descending artery Red arrow pointing at the dilated left anterior descending artery measuring 0.35 cm

**Figure 3:**
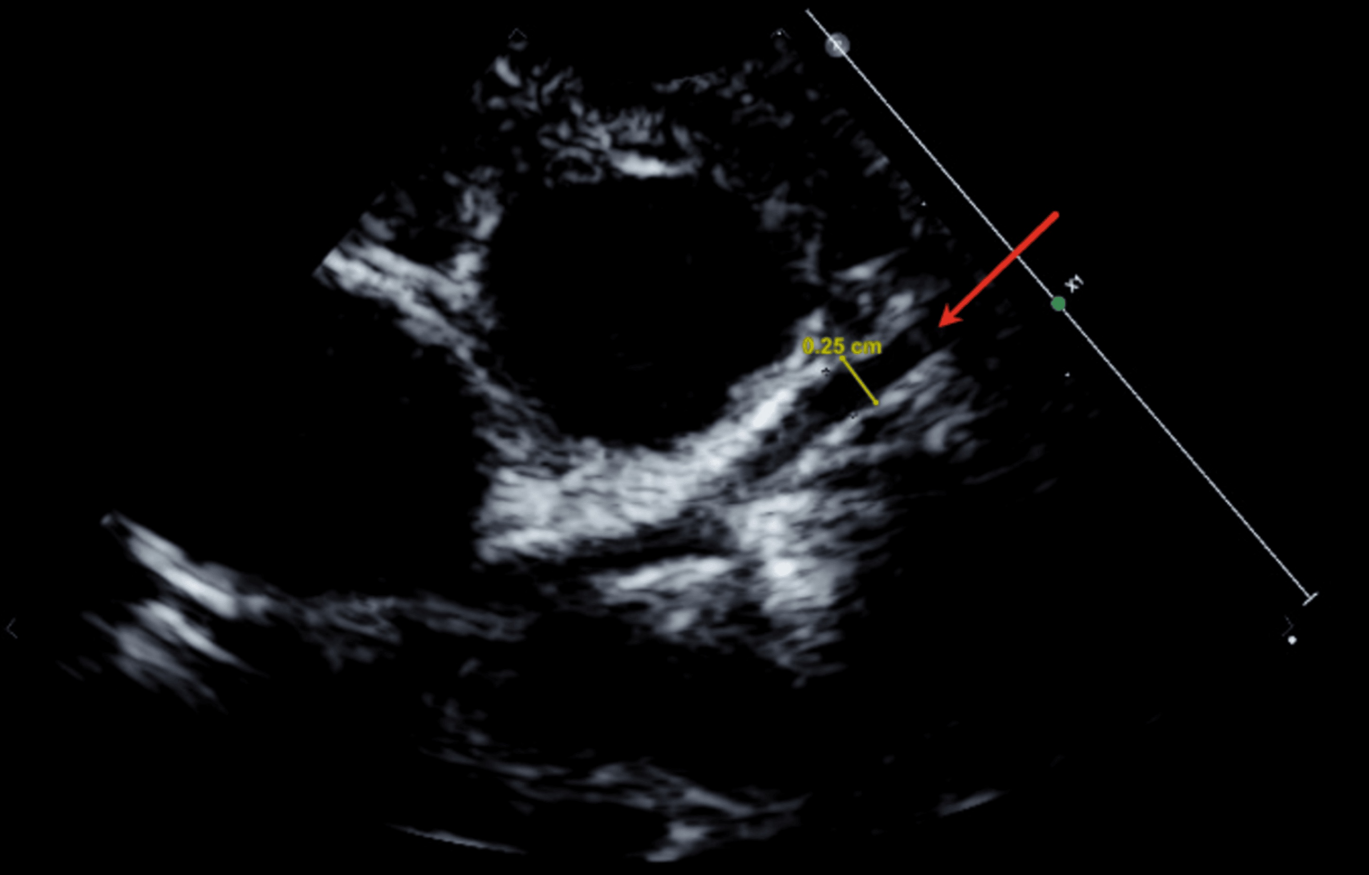
Echocardiogram from the parasternal short-axis view showing a dilated right coronary artery Red arrow pointing at a dilated right coronary artery measuring 0.25 cm

An electrocardiogram (ECG) showed sinus tachycardia with mild left ventricular hypertrophy. It was then decided to complete the evaluation with an echocardiogram that revealed dilated coronary arteries (Figures 2, 3), aortic root dilatation, and a high aortic flow rate with thrombocytosis, suggestive of late-term complications of KD. The specific measurements were as follows: Left anterior descending artery 0.35 cm (Figure 2) and right coronary artery 0.25 cm (Figure 3), indicating severe coronary dilatation. It was associated with a patent foramen ovale with left-to-right shunt and trivial aortic regurgitation.

The child was diagnosed with atypical KD and started on high-dose aspirin (50 mg/kg/day divided into four doses) along with IVIG (2g/kg once) and methylprednisolone (10 mg/kg/day for three days) and was tapered to 0.5-1 mg/kg daily for four weeks. After 48-72 hours of treatment, she began to show improvement. She was discharged with a prescription for oral aspirin (5mg/kg/day) and oral prednisolone for four weeks with a tapering protocol.

A week later, the child presented to the ER due to vomiting and passing blood in her stool for three days, accompanied by noticeable irritability. She was also observed holding her legs close to her abdomen. Laboratory investigations showed leukocytosis, anemia, thrombocytosis, and elevated inflammatory markers including high CRP, ESR, and procalcitonin (Table 2).

**Table 2:** Laboratory findings during the second admission

Test	Day 1	Day 2	Day 14	Day 16	Normal values
White blood cell count	30.90 × 10^9^/L (high)	14.36 × 10^9^/L (high)	15.55 × 10^9^/L (high)	15.54 × 10^9^/L (high)	4.0 - 11.0 × 10^9^/L
Hemoglobin	7.30 g/dL (low)	7.00 g/dL (low)	7.60 g/dL (low)	8.00 g/dL(low)	12.0 - 16.0 g/dL
Hematocrit	25.90 % (low)	24.60 % (low)	25.20 % (low)	27.80 % (low)	36- 46%
Platelet	826 × 10^9^/L (high)	693 × 10^9^/L (high)	683 × 10^9^/L (high)	754 × 10^9^/L (high)	150 -450 × 10^9^/L
C-reactive protein	7.2 mg/dL (high)	23 mg/dl (high)	93 mg/dL (high)	160 mg/dL (high)	< 3.0 mg/dL
Procalcitonin	-	-	-	0.23 ug/L (high)	< 0.05 µg/L
Erythrocyte sedimentation rate				23mm/h (high)	0-10 mm/h

**Figure 4:**
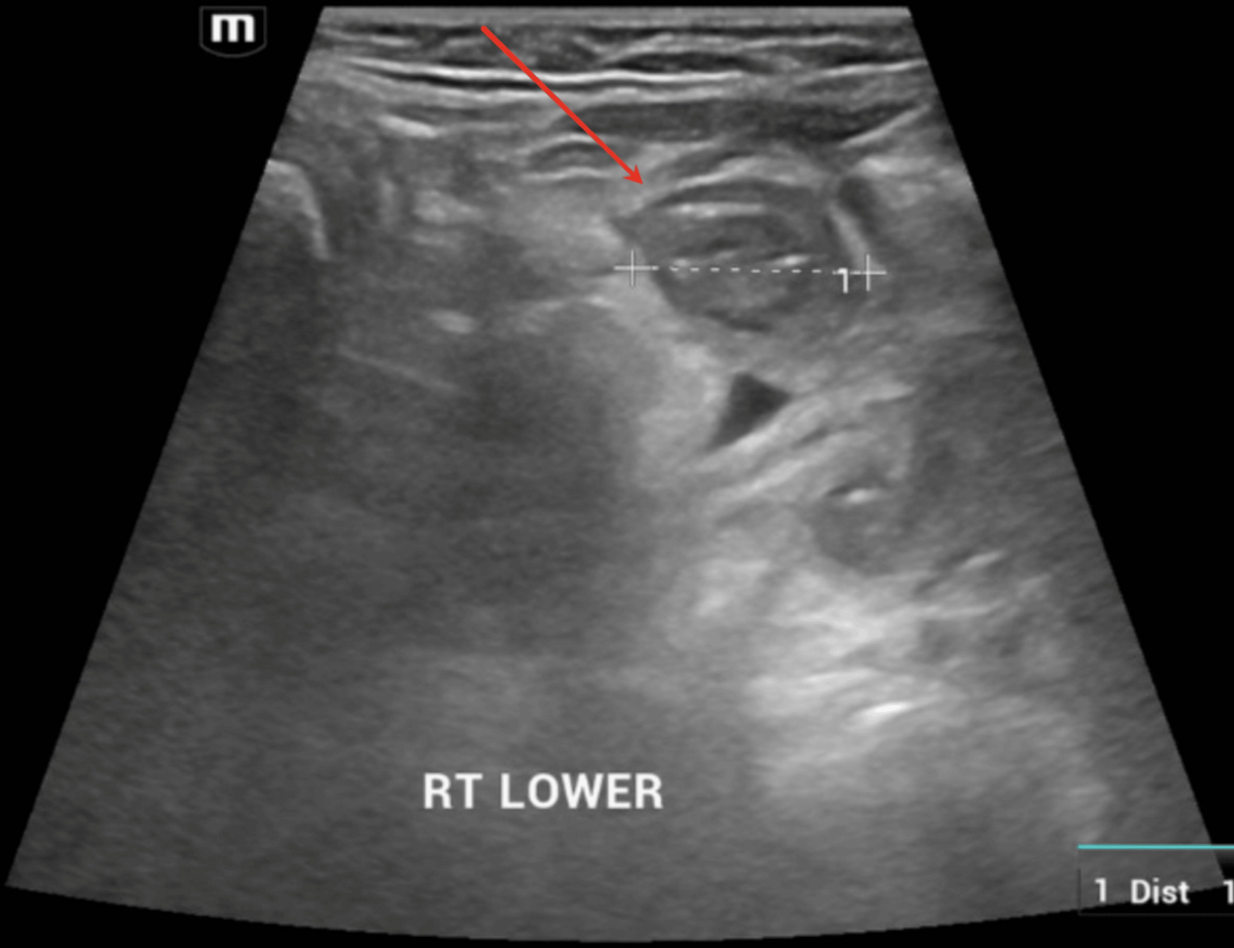
Ultrasound abdomen transverse view showing a doughnut sign Red arrow pointing at the doughnut sign

**Figure 5:**
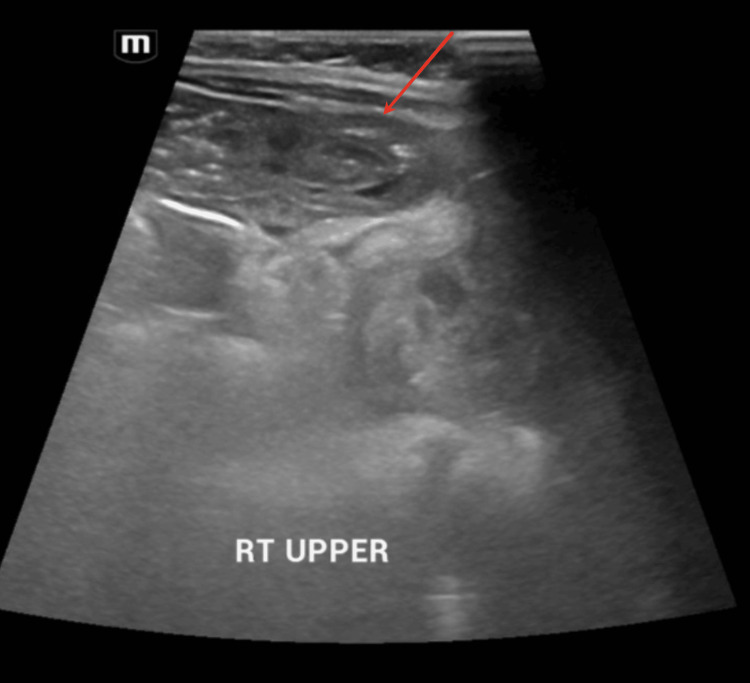
Ultrasound abdomen longitudinal view showing a pseudokidney appearance Red arrow pointing at pseudokidney appearance

Abdominal ultrasound performed revealed a newly developed doughnut-shaped mass measuring 1.4 x 1.5 x 1.8 cm on the right side of the abdomen (Figure 4). A pseudokidney appearance could also be appreciated (Figure 5). Both ultrasound findings were strongly suggestive of intussusception and she was diagnosed with transient intussusception. She was managed conservatively without the need for surgical intervention after a discussion with the pediatric surgical team.

She continued receiving aspirin (5mg/kg/day) and was started on IV metronidazole and symptomatic treatment. During her hospital stay, the patient improved and was discharged with a follow-up in the pediatric cardiology clinic. During her follow-up at two weeks, she was well, happy, and playful. There was improvement in her coronary dilatation and the cardiology team arranged further follow-up.

## Discussion

KD is a rare but serious systemic vasculitis involving medium- and small-sized arteries in early childhood (i.e., age six months to five years) and can lead to severe cardiovascular complications such as coronary artery aneurysms in 25% of untreated patients, which can be reduced to 4% with appropriate treatment [7]. The exact cause of KD is unknown, but it is thought to involve an abnormal immune response, possibly triggered by an infectious agent in genetically predisposed individuals. The diagnostic criteria of KD are based on clinical findings, and more than one-half of affected children meet the diagnostic criteria. However, the remainder of patients present with a variety of atypical clinical manifestations that may involve one or more organ systems [8]. KD shows epidemiological variations, with a higher incidence in Asian populations, especially in Japan [9], with the primary age of patients affected by KD being between two and three years of age [10]. Although KD is a clinical diagnosis and laboratory investigations have a minimal role in the diagnosis, children may have elevated inflammatory markers like CRP and ESR, thrombocytosis, and neutrophilic leukocytosis [11].

Incomplete or atypical KD tends to occur in children younger than six months or older than five years, and they are generally at higher risk of coronary artery aneurysms and IVIG resistance. Incomplete KD is suspected when fewer than four criteria are met in a child with prolonged fever. Additional investigations and findings that support the diagnosis include high inflammatory markers (CRP and/or ESR), leukocytosis, anemia, thrombocytopenia, and echocardiogram findings [12]. The current diagnostic criteria for KD do not include imaging findings per se. However, anecdotal reports have indicated that imaging findings were helpful in the early diagnosis of KD, regardless of whether patients present with typical or atypical disease. We believe that imaging should be incorporated into future guidelines to facilitate diagnosis before the criteria are fully evident to ensure timely management. This case is notable as it highlights an unusual occurrence of KD in a child younger than the typical age of onset, presenting with unusual clinical manifestations involving mainly the gastrointestinal system which occurs in about 25-30% of patients with KD [13]. There have been scarce cases documented in the literature with similar presentations, for instance, Idris et al. described a four-month-old girl with incomplete KD [14], while Hussain and Ruiz reported a three-year-old boy who presented with intussusception prior to developing the hallmark presentation of KD [15].

The patient’s readmission after initial treatment highlights the potential for recurrent gastrointestinal symptoms and complications, such as transient intussusception, which has been reported in association with KD [16]. This multisystem involvement occurs because the inflammatory process of KD extends into the arteries and veins of organs throughout the body during the acute phase of the illness, as has been demonstrated in many pathologic reports. The use of imaging modalities, such as ultrasound and CT, is crucial for accurately diagnosing and managing complications, with recent literature highlighting their importance in identifying gastrointestinal complications related to KD that may not be apparent at first sight [17]. 

Coronary artery abnormalities are a significant complication of KD, occurring in approximately 25% of untreated patients. It is the leading cause of acquired heart disease in developed nations and is gradually surpassing rheumatic heart disease in developing countries [18]. In our case, echocardiographic findings revealed dilated coronary arteries and aortic root dilation, indicative of late-term complications of KD. Imaging, especially echocardiography, is vital for assessing coronary artery involvement, as coronary artery aneurysms are a severe complication of KD. Early treatment with IVIG and aspirin reduces the risk of these abnormalities [19]. Serial echocardiograms are performed during the acute phase and follow-up to monitor coronary artery size and function which can be utilized to monitor disease progression [17].

## Conclusions

The diagnosis of KD is based on a combination of clinical features; no pathognomonic test is currently available. This case emphasizes the need for heightened awareness of KD in young patients, particularly when classical symptoms are absent. The unusual presentation in this six-month-old girl marked by gastrointestinal symptoms led to a delayed diagnosis. Given these potential clues, a pediatrician should consider KD in the differential diagnosis when irritability persists with high inflammatory markers, especially if accompanied by any cardiovascular or gastrointestinal symptoms. Prompt identification and treatment with intravenous immunoglobulin and aspirin are crucial in managing the serious cardiovascular complications associated with KD. Additionally, the recurrence of gastrointestinal issues highlights the potential for further complications, like transient intussusception, necessitating vigilant follow-ups and comprehensive care. Follow-up with a multidisciplinary team, including a pediatrician, pediatric cardiologist, and pediatric rheumatologist, is essential in the management of KD to monitor the effectiveness of treatment and prevent complications. This case contributes to the growing body of literature on atypical KD presentations and underscores the importance of thorough clinical evaluation in pediatric patients. 
